# How the public uses social media wechat to obtain health information in china: a survey study

**DOI:** 10.1186/s12911-017-0470-0

**Published:** 2017-07-05

**Authors:** Xingting Zhang, Dong Wen, Jun Liang, Jianbo Lei

**Affiliations:** 1 Peking University Third Hospital, Beijing, China; 2 IT Center, Second Affiliated Hospital Zhejiang University School of Medicine, Hangzhou, China; 3 Center for Medical Informatics, Peking University, 38 Xueyuan Rd, Haidian District, Beijing, 100191 China; 4 School of Medical Informatics and Engineering, Southwest Medical University, Luzhou, China

**Keywords:** Social media, WeChat, Health education, Health information acquisition

## Abstract

**Background:**

On average, 570 million users, 93% in China’s first-tier cities, log on to WeChat every day. WeChat has become the most widely and frequently used social media in China, and has been profoundly integrated into the daily life of many Chinese people. A variety of health-related information may be found on WeChat. The objective of this study is to understand how the general public views the impact of the rapidly emerging social media on health information acquisition.

**Methods:**

A self-administered questionnaire was designed, distributed, collected, and analyzed utilizing the online survey tool Sojump. WeChat was adopted to randomly release the questionnaires using convenience sampling and collect the results after a certain amount of time.

**Results:**

(1) A total of 1636 questionnaires (WeChat customers) were collected from 32 provinces. (2) The primary means by which respondents received health education was via the Internet (71.79%). Baidu and WeChat were the top 2 search tools utilized (90.71% and 28.30%, respectively). Only 12.41% of respondents were satisfied with their online health information search. (3) Almost all had seen (98.35%) or read (97.68%) health information; however, only 14.43% believed that WeChat health information could improve health. Nearly one-third frequently received and read health information through WeChat. WeChat was selected (63.26%) as the most expected means for obtaining health information. (4) The major concerns regarding health information through WeChat included the following: excessively homogeneous information, the lack of a guarantee of professionalism, and the presence of advertisements. (5) Finally, the general public was most interested in individualized and interactive health information by managing clinicians, they will highly benefit from using social media rather than Internet search tools.

**Conclusions:**

The current state of health acquisition proves worrisome. The public has a high chance to access health information via WeChat. The growing popularity of interactive social platforms (e.g. WeChat) presents a variety of challenges and opportunities with respect to public health acquisition.

## Background

The rapid development of the Internet and new media has remarkably changed people’s lifestyles. The number of global Internet users has exceeded 3 billion, and the Internet penetration rate reached 42% [1]. According to the Pew Research Center, 84% of American adults have access to the Internet [2]. Furthermore, 72% of online American adults use Facebook [3]. According to ‘The 36th Statistical Report on Internet Development in China’, from June 2015, the number of Chinese netizens reached 668 million, mobile Internet users reached 594 million, and the percentage of Internet surfing via mobile was as high as 88.9% [4].

Similar to Facebook, WeChat, a free application released in 2011 by Tencent, Inc., has become the most widely and frequently used social networking platform in China. WeChat provides many services for daily living, including instant messaging, free phone calls, interest or private groups, browsing and posting for information sharing on moments, mobile payments, sending red envelops, etc. [5]. According to the latest data from Tencent, Inc., the number of active monthly customers reached 700 million in March 2016, from 200 countries and in nearly 20 different languages; 61% of WeChat users open WeChat more than 10 times per day, 36% open WeChat more than 30 times per day, and 32% of users used WeChat for longer than 2 h per day. Additionally, 61.4% of users check moments each time they open WeChat; and 28% of users have more than 200 friends, which is more than twice that of last year’s data. In April 2016, the time spent, daily, on mobile Internet averaged 200 min per user, and WeChat accounted for 35% of that time [1]. Furthermore, 200 million users linked WeChat with credit cards, 70% of users spend more than 100 RMB (local currency in China) per month on WeChat, and 8 billion RMB were sent via WeChat Red Envelopes during the Spring Festival of 2016.

WeChat is more than just an innovative mobile application installed on over 90% of smartphones and integrated into most people’s routines: it is now an inevitable tool transforming daily life of users in many ways. As part of a new lifestyle, WeChat spearheads a new era of mobile Internet communication [6]. Most importantly, a variety of health-related information was continuously generated and transmitted among tremendous numbers of users through the different functions of WeChat, providing WeChat with enormous potential to affect the general public’s health status. Apparently, research on how social media transforms the public’s health status is forecast to be on rapid rise and is highly necessary.

Previous studies [7, 8] have focused on health information acquisition via the Internet and new media, as well as the related improvement of healthy living habits. According to the ‘Pew Internet and American Life Project,’ 61% of American adult netizens actively acquired health-related information online. Moreover, almost 60% of respondents admitted that Internet health information impacted their health management strategy [9]. The information acquired via the Internet is primarily associated with areas such as health care, disease symptoms, and treatments [10]. A cancer information survey among Chinese people (Institute of Public Opinion, Renmin University of China) reported that in Beijing and Hefei, 76.3% of computer-using respondents acquired health information via the Internet, while 68.8% of mobile terminal-using (e.g. smartphone and iPad) respondents acquired health information from the Internet. With an increasing sense of health among the general public, new media’s popularity as a means of acquiring health-related information has also grown [11]. Social media-with material contributed by patients themselves-provides an opportunity to better understand views on health care from a patient’s perspective [7]. Furthermore, social media platforms emphasize a more customer-oriented experience and enables the sharing of health information [12], and a previous study showed that social media usage has positive effects on health behaviors [13].

Although health information acquisition and usage via new media has been studied in foreign countries, few studies have been conducted in China. Furthermore, the health information services provided by professional institutions, hospitals, and communities are greatly insufficient. As the rapidly emerging and most prominent social media in China, WeChat plays an important part in modern lifestyles. Whether and how this trend would affect the health status of the general public is unknown. This study was the first quantitative attempt to investigate how the general public acquires health education, especially the current availability, problems, and tendency of health education using social media WeChat. Further functions developed based on our study may greatly promote the efficiency of health education and spread insight across the global community.

## Methods

### Samples

As the most widely and frequently used social communication tool for general public in China, WeChat has a powerful friends network: 57.3% of customers made new friends, or got back in touch with old friends via WeChat [5]. This makes it possible to administer questionnaires via WeChat using a convenience sampling method. From October 10–21, 2015, the self-designed questionnaire on health education recognition was sent to 2000 users and WeChat groups (approximately 20 chat groups of between 8 and 314 participants) from the author’s WeChat account. Each receiver was requested to forward the questionnaire link to their own friend’s network. A total of 1739 questionnaires were collected, with 1636 determined valid.

### Questionnaire preparation and survey method

The questionnaire was produced, distributed, and collected with the online survey tool Sojump (http://www.sojump.com). Sojump is a professional online survey, evaluation, and voting platform that permits humanized services including questionnaire design, data collection, custom reporting, and result analysis.

The questionnaire titled “questionnaire on knowledge, attitude, and behavior of health education via WeChat” was self-designed based on literature review [14–16], the needs of this study, and the researchers’ experiences. As a result, 6 major topics were developed in the questionnaire: demographic indexes, current health education status, WeChat use, WeChat use for health education, expected health education approach, and problems related to medical and health information via WeChat. Then, we built a series of measurement indicators, including channels, frequencies, overall evaluation, satisfaction of searching for health information, etc. These indicators regarding the use of WeChat to acquire health information were categorized into 3 levels, from large to small: type, means, and mode. Finally, we set up a variety of questions, which included short answer, multiple choices, rating questions, and introduced Likert scaling and percentages for quantification.

Once the questionnaire design was finished, 5 participants, 2 of whom were survey experts, conducted a pilot test. Then, the research team reworked the questionnaire to address concerns that arose during the pilot. These were in regards to response time, question design, question order, and question content.

The long-term questionnaire link is http://www.sojump.com/jq/8965372.aspx. To improve the quality of questionnaire recycling and collect as many valid questionnaires as possible, electronic red envelopes of varying value were randomly distributed, thereby encouraging respondent initiative; the actual value was 1 RMB for everyone.

### Statistical analysis

Questionnaires were analyzed to eliminate invalid questionnaires. The exclusion criteria included repetitive IP addresses, logic errors, same answers for consecutive questions, and questions left blank. Finally, a total of 103 questionnaires were excluded, resulting in 1636 valid questionnaires. Data analysis was performed using Sojump and Excel, SPSS20.0.0. Descriptive analysis was conducted automatically via the statistical functions of Sojump. Lastly, an ANOVA test was performed via SPSS to explore the impact of different demographic characteristics of the respondents on the scores of WeChat health information quality and health education patterns.

## Results

### General information of respondents

**Fig. 1:**
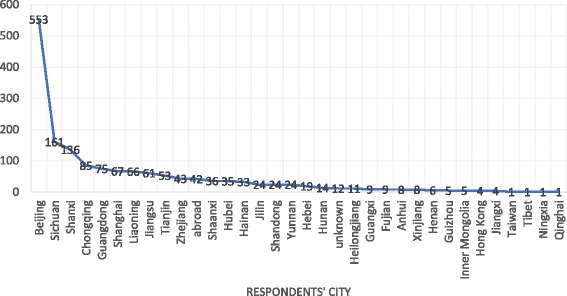
Geographical Distribution

The 1,636 respondents (male 784, female 852) were from 32 provinces and cities across the country. Age ranged from 18 to 50 years old, in a uniform distribution, and 90% of respondents possessed college/university education or above (see Table 1.). The majority of respondents were from Beijing, Sichuan, Shanxi, Chongqing, Guangdong, Shanghai, Liaoning, Jiangsu, and Tianjin (Fig. 1). All questionnaires were submitted through smartphones.

**Table 1:** General information of respondents

Category	Number of Respondents	Percentage (%)
Gender		
Male	784	47.92
Female	852	52.08
Age		
Under 18	11	0.67
18–25	309	18.89
26–30	301	18.40
31–40	499	30.50
41–50	443	27.08
51–60	69	4.22
Above 60	4	0.24
Education		
Primary school	8	0.49
Middle school	21	1.28
High school/Vocational school/Secondary school	89	5.44
College/University	886	54.16
Master	505	30.87
Doctorate or above	127	7.76
Total	1636

### Current state of health education

**Fig. 2:**
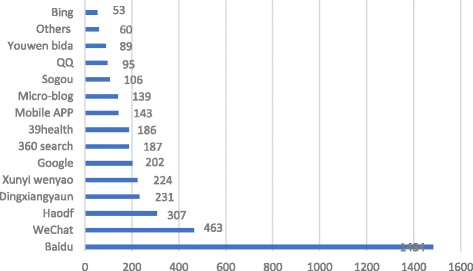
Health Information Search Channels

Most respondents acquired health education via the Internet (71.94%), and nearly one-third of them frequently searched online for health information (often 26.41%, always 3.61%). As shown in Fig. 2, Baidu was the top choice for online searches (90.71%), and WeChat was second (28.30%).

The general public’s major concerns regarding existing health education included lack of timely attention (58.62%), insufficient individualized attention (68.09%), and poor interactions (68.34%). Only 12.41% of the respondents were satisfied with the results provided on health information via the Internet; moreover, only 6.61% were generally satisfied with the current health education system in China (see Table 2).

**Table 2:** Overall Assessment

	Overall assessment of searching for medical knowledge via internet	Overall assessment of current health education system
Very poor	71 (4.34%)	124 (7.58%)
Worse	293 (17.91%)	489 (29.89%)
Normal	1069 (65.34%)	915 (55.93%)
Good	186 (11.37%)	103 (6.30%)
Excellent	17 (1.04%)	5 (0.31%)

### WeChat usage

**Fig. 3:**
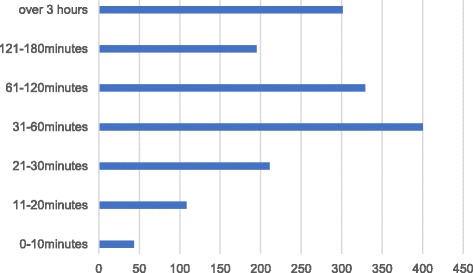
Time Using WeChat Each Day

As other statistics show, our study participants used WeChat frequently: 97% used WeChat every day, half of the respondents opened it at least 20 times a day, and 16.01% opened it as much as 40 to 50 times a day (see Table 3). Over half of the respondents stayed on WeChat for at least 1 h each day, while 18.97% used it for more than 3 h (Fig. 3).

**Table 3:** Total Times Checking WeChat Each Day

Number of times checking WeChat each day (times)	1–10	11–20	21–30	31–40	41–50
Total	401 (25.27%)	445 (28.04%)	314 (19.79%)	173 (10.9%)	254 (16.01%)

### Current state of health information via WeChat

WeChat is a potentially powerful means of providing health education. Seeing health information via WeChat is common. 98.35% of respondents reported that they have seen health information, while 29.71% of respondents often or always see health information; 97.68% of respondents reported that they have opened and read health information, and 32.33% of respondents open and read health education articles regularly (see Table 4). The primary means of receiving health information were through Moments (70.54%), public accounts (53.36%), and group chats (31.17%), which are major functions of WeChat. (WeChat suggests users to post images and text, share music and articles, as well as comment and “like” in the Moments; WeChat also supports users in registering as a public account, which enables them to push feeds to subscribers, interact with subscribers, and provide them with services).

**Table 4:** Frequency of Accessing WeChat Medical Articles

	Frequency of seeing a medical article via WeChat	Frequency of opening and reading a medical article
Never	27 (1.65%)	38 (2.32%)
Occasionally	547 (33.44%)	498 (30.44%)
Sometimes	576 (35.21%)	571 (34.9%)
Often	453 (27.69%)	474 (28.97%)
Always	33 (2.02%)	55 (3.36%)

An evaluation of the quality of health information on WeChat revealed that readability had the highest score (63.83), whereas professional aspects and health benefits received lower scores (55.28 and 55.23, respectively). 41.32% (agree and strongly agree) of respondents believed that WeChat health information had no significant impact, 44.25% of respondents were neutral, and only 14.43% believed that WeChat health information could improve health (see Table 5). In addition, more than 60% of respondents were concerned that there were many problems about health knowledge in WeChat, such as unprofessional information, too much similar information, too many advertisements, etc., and readability was considered the least significant problem (see Table 5).

**Table 5:** Potential problems of WeChat health information

	Strongly disagree	Disagree	Neutral	Agree	Strongly agree
Not professional enough	53 (3.24%)	61 (3.73%)	399 (24.39%)	701 (42.85%)	422 (25.79%)
Readability	52 (3.18%)	245 (14.98%)	682 (41.69%)	514 (31.42%)	143 (8.74%)
Too much similar information	38 (2.32%)	48 (2.93%)	426 (26.04%)	808 (49.39%)	316 (19.32%)
Too many advertisements	38 (2.32%)	68 (4.16%)	414 (25.31%)	642 (39.24%)	474 (28.97%)
No significant impact on improving health	43 (2.63%)	193 (11.8%)	724 (44.25%)	507 (30.99%)	169 (10.33%)

### Ideal health education mode expected

**Fig. 4:**
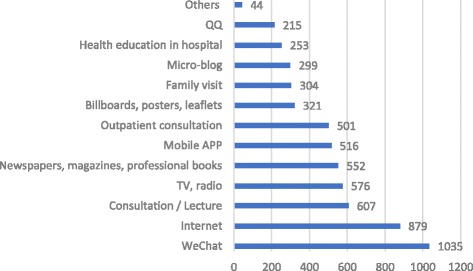
Most Expected Health Education Type

**Fig. 5:**
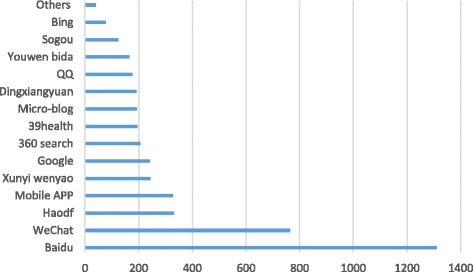
Most Expected Means for Searching for Health Information

Study participants held high expectations for new media like WeChat. When asked about the most expected means for obtaining health education, WeChat was chosen as the most popular with a selection rate of 63.26%, which was higher than the selection rate of 53.73% for the Internet, which was ranked second (see Fig. 4). When asked about the most expected means for searching health information, Baidu, a traditional means to obtain health information, was the most popular (80.20%), followed by WeChat (46.76%). Both were much higher than other search engines, professional websites, and new media such as haodf.com (20.23%), mobile applications (19.99%), and xywy.com (14.85%) (see Fig. 5).

The concept of health education is more general than that of searching for health information. The latter primarily involves search engines in which an active mode is crucial, while the former has broader meaning. Social media may play broader and more important roles by sharing or pushing individualized health education information to target consumers, while Baidu is just a search engine. When asked about the most expected mode of health education within WeChat, ‘personalized health education information from the doctor in charge of you, and reminders for you to read’ was the highest rated item (with a score of 76.42) among survey participants (see Table 6). This indicates that interactive and individualized health education by a managing physician is crucial, since it would remarkably increase the chance of reading health information related to the consumers’ personal health status, and thus help improve the effectiveness of health education. More personalized solutions may be developed and evaluated to meet such needs of the general public.

**Table 6:** Most expected mode of health education

Health education mode	Medical and health information from friends’ moments, group chats, public account sharing (open to all)	Normal health education information from the doctor in charge of you	Personalized health education information from the doctor in charge of you	Personalized health education information from the doctor in charge of you, and reminders for you (for example, referred to you, @ you, private letter to you)
Score	61.40	70.15	74.15	76.42

### SPSS statistical analysis

An ANOVA test was performed on the readability, usefulness, and professionalism scores of WeChat health information quality. The test showed that the feedback on quality of WeChat health information had statistically significant differences in age and educational background. Respondents older than 60 years old rated the lowest score (22.25), however, there were only 4 samples in this age range; scores from other age ranges were similar. Respondents from middle school scored highest, however, this sample size was also too small (only 21); scores from other educational backgrounds were similar. In addition to usefulness, both readability and usefulness differed with gender, with males scoring 2–3 points higher than females (95% CI, *P* < 0.05). Regarding the best health education mode, there were statistically significant differences in both age and educational background. However, the difference across age was due to the small sample size of respondents older than 60 years old. Respondents from different educational backgrounds scored differently on third and fourth health education means, showing that the higher the level of education, the higher score (95% CI, *P* < 0.05).

## Discussions

### Generally unsatisfactory health education

Health education represents an important country-level public health effort. However, the current status of health education in China is disappointing. Traditional health education modes have been gradually replaced by new technologies. This study shows that the Internet has become the primary source of health education. Nearly one-third of the study participants used the Internet to search for health information regularly, and Baidu was the primary search engine utilized. However, only 12% of respondents were satisfied with their experience searching for information via the Internet, which may be partly associated with Baidu’s commercial policies and ranking rules, where information is provided or ranked based on how much a user pays Baidu. Overall satisfaction with current health education was even worse, with only 6% of people satisfied.

In China, people’s health awareness is generally poor. The majority have a weak sense of how to take care of themselves and/or an ability to do so. Furthermore, harmful lifestyles are very common. Consequently, health education in China is a formidable task. Medical and healthcare workers are the primary sources of health education, yet there is a gap between health education and clinical services due to high clinical workloads, busy schedules of clinicians, and major clinical service goals focused only on curing patients. Current health education ignores the true demands of the general public. It lacks a focus on individual needs and humanistic concerns, resulting in poor interactions and effectiveness. The popularity of the Internet and rise of new media greatly challenges the traditional health education mode, yet also provides new opportunities for innovation [17].

### WeChat as an essential pathway for health information acquisition

This study predicted an increase in health education information acquisition via new media. The Internet and WeChat are the most popular choices for acquiring health information. Nearly one-third of the general public often receive health information, and nearly one-third often open and read health information through WeChat moments, public accounts, or group chats. A survey study by Hou et al. [18] showed that 68.7% of respondents from Beijing citizens followed WeChat public accounts, micro-blogs, or often browsed health-related web pages. According to the Chinese medical and health industry public data insight report [19], in the fourth quarter of 2015, 35.4% of headline articles from health-related public accounts had more than 10,000 readings, with an average 13,223, reflecting a high attention to health information by WeChat users. The study on health information acquisition channels by Zhang et al. [11] also suggested that passive acquisition through WeChat moments was an important medium for college students. For some, it was the only means by which they obtained health information. WeChat moments, public account notifications, and group chats are the 3 main channels for information acquisition. In contrast, WeChat search, mobile QQ browser, WeChat hot article search, and Sogou WeChat search functions are utilized less frequently. These results indicate that passive acquisition is more frequent than active acquisition [20].

WeChat is widely accepted throughout China due to its numerous interactive functions. Currently, the number of WeChat customers has exceeded 900 million, with 150 million customers staying online for at least 2 h every day [5]. With a good user base, WeChat can be used to precisely send information to target populations. WeChat information transmission starts from acquaintances with relatively stable relationships, and expands to strangers, building trust between senders and receivers [21]. WeChat communication is characterized by dual-direction and interaction. Users are able to send health information according to the receiver’s taste, such as via text, voice, images, and video. In the future, different notifications could be sent according to individual preference, which could satisfy the public’s increasing health education requirements and provide a solid basis for health information dissemination [22].

### Integrate WeChat into the ideal health education mode

WeChat represents a more convenient and accessible tool for health education in China. Health information acquisition via WeChat is more convenient, timely, cost-effective, privacy protective, and avoids embarrassment. Furthermore, the technical development of big data and the Internet of Things allow individuals to access and track effective health information and customize on-demand health information. Thus, to a certain extent, WeChat contributes to greater freedom regarding individual health decisions. An increase in health knowledge via WeChat information acquisition increases the public’s health decision-making and action ability [23]. It also helps break the traditional imbalance between doctors and patients, and promotes efficient communication between them, thereby saving public medical resources.

Therefore, autonomous health education via WeChat is a useful tool to address the current shortcomings in health education. The present study also shows that WeChat and other new media are the most accepted health education channels. However, only 14.43% of our respondents believed that WeChat health information could help their health, reflecting that the public is suspicious about the quality of WeChat health information. A survey Study by Hou et al. [18] showed that more than half of the responding Beijing residents believed that Internet health information was not reliable. In terms of the most-wanted health education mode through WeChat, the item with the highest score was ‘personalized health education information from the doctor in charge of you, and reminders for you to read’. Since the patient’s primary doctor is familiar with their condition, this mode promotes well-directed health education. In addition, because of the communication characteristics of WeChat, doctor-patient communication could be enhanced and ultimately more effective.

### Limitations

First, the study adopted a convenience sampling method that started from a personal WeChat account. Although respondents were sampled randomly across the country and participation was voluntary, the sample number was too small to represent the general public (all WeChat users). Fortunately, the distribution of the questionnaire was very powerful: 1,636 valid replies were received, and respondents were distributed over 32 provinces and cities across the country. Thus, our sample was representative to certain degree. Furthermore, the age range distribution was concentrated between 18–50, and the numbers of respondents in the age groups of 18–25, 26–30, and 31–40, were almost evenly distributed. This represents the main core of active WeChat users. According to a large-scale survey, the average age of WeChat users is only 26 years old, 97.7% of users are under the age of 50, and 86.2% of users are 18–36 years old [24]. The purpose of this study was not to draw upon the general characteristics of the WeChat population but to obtain knowledge of social media on health information acquisition through the typical population group so that we can learn how to deal with the future development of social media for health education and information acquisition. Another limitation is that the questionnaires were distributed online, where there were many uncontrollable factors. In addition, since the required questionnaire completion time was short, the results possibly contained a degree of randomness. These above factors might have caused bias. Lastly, to conduct a more accurate and convincing study, more samples are required, along with greater attention to detail in controlling variables and improving questionnaire evaluation.

### Future directions

There were major concerns with the quality of health education information on WeChat, especially with regards to content. WeChat uses open information generation and communication modes, with limited control of the health information made available. Therefore, it is essential to evaluate article quality, and improve dissemination of high quality articles. Another important direction is to provide directed assistance so that patients can obtain health data and develop individualized plans for health education. WeChat could also develop functions that include new online medical services, patient-doctor communication, and patient-to-patient communication. Finally, areas such as supervision of WeChat health information, moral standards, and privacy, should be considered.

## Conclusions

This study is the first quantitative investigation of people’s view on health information via WeChat. Results showed that the current status of health education is worrisome and unable to meet the demands of the general public. Although there are many opportunities to access health information, the public is highly suspicious of the professionalism of the health information they see from WeChat. This study predicted that individualized health education based on WeChat, in which a wide variety of health topics is shared, commented on, and discussed, could play a central role in the future of health education efforts in China because WeChat is well integrated into society.
